# Iron Metabolism in Thalassemia and Sickle Cell Disease.

**DOI:** 10.4084/MJHID.2009.006

**Published:** 2009-10-27

**Authors:** Raffaella Mariani, Paola Trombini, Matteo Pozzi, Alberto Piperno

**Affiliations:** Department of Clinical Medicine and Prevention, University of Milano-Bicocca and Centre for Diagnosis and Treatment of Hemochromatosis, S. Gerardo Hospital, Monza, Italy

## Abstract

There are two main mechanisms by which iron overload develops in thalassemias: increased iron absorption due to ineffective erythropoiesis and blood transfusions. In nontransfused patients with severe thalassemia, abnormal dietary iron absorption increases body iron burden between 2 and 5 g per year. If regular transfusions are required, this doubles the rate of iron accumulation leading to earlier massive iron overload and iron-related damage. Iron metabolism largely differs between thalassemias and sickle cell disease, but chronic transfusion therapy partially normalize many of the disparities between the diseases, making iron overload an important issue to be considered in the management of patients with sickle cell disease too. The present review summarizes the actual knowledge on the regulatory pathways of iron homeostasis. In particular, the data presented indicate the inextricably link between erythropoiesis and iron metabolism and the key role of hepcidin in coordinating iron procurement according to erythropoietic requirement. The role of erythropoietin, hypoxia, erythroid-dependent soluble factors and iron in regulating hepcidin transcription are discussed as well as differences and similarities in iron homeostasis between thalassemia syndromes and sickle cell disease.

## Introduction:

In humans total body iron stores is maintained normally within the range of 200–1500 mg (in men, the normal concentration of iron in the storage pool is 13 mg/Kg and in women 5 mg/kg) by adequate adjustment of intestinal iron absorption, since no excretory mechanisms exist^1^. Iron overload is a common complication of thalassemia syndromes which could lead per se to the development of organ damage and increased mortality^2^. There are two main mechanisms by which iron overload develops in thalassemias: increased iron absorption due to ineffective erythropoiesis and blood transfusions. Due to the different physiopathology of anemia in thalassemia and sickle cell disease (SCD), and the different indications in the use of transfusions in thalassemia major, intermedia, and SCD, there are significant differences in the physiopathology of iron overload and iron-related complications in these disorders.

## Iron metabolism:

### 

#### Body iron pathways

**Figure 1.**
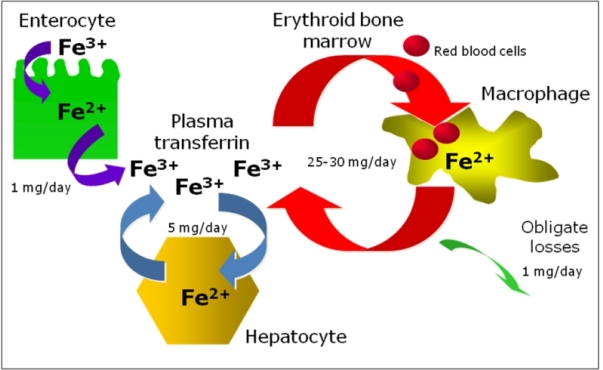
Body iron homeostasis.

Because there is no known regulated mechanism for iron excretion, and the amount of iron entering the body each day represents less than 0.1% of the total body iron endowment, most circulating iron must be derived from the recycling of iron already within the system^3^ (Figure 1). From a quantitative point of view, the most important pathway of iron metabolism is the unidirectional recycling of iron from senescent red blood cells to the erythroid bone marrow through macrophages^1^,^4^. Renewed interest has been developed on the role of macrophages within the erythroblastic islands, the specialized niches in which erythroid precursors proliferate, differentiate, and enucleate in the bone marrow^5^. These hematopoietic subcompartments are composed of erythroblasts surrounding a central macrophage. It was suggested that the macrophage functions as a “nurse” cell, providing iron to developing erythroblasts for heme synthesis^6^. An attractive hypothesis proposed for central macrophages is that ferritin, synthesized by macrophages, secreted via exocytosis, and subsequently taken up by erythroblasts could be a source of iron for heme synthesis^7^. It is not known whether central macrophages might release iron also by the ferroportin-dependent exporting pathway and further studies are needed to clarify all this matter.

The second pathway is the cycling of iron from hepatocytes to the blood and viceversa, according to body needs and the third is iron absorption through duodenal and upper jejunum that balances the 1–2 mg daily iron loss occurring through cellular exfoliation (see below: iron absorption and iron storage paragraphs).

### Iron transport and utilization:

The systemic transporter of iron in the body is transferring, an abundant, high affinity iron-binding protein. Under normal circumstances, TF carries nearly all serum iron collected from duodenal absorptive epithelium, macrophages and hepatocytes, and dampens its reactivity. Very small amounts of iron may be loosely associated with albumin or small molecules. In normal human subjects, iron occupies approximately 30% of the iron-binding sites on plasma TF^1^. The saturation of TF by iron shows diurnal fluctuation exhibiting a morning peak and an evening nadir^8^. It is likely to be higher in the portal circulation, where recently absorbed iron from the intestine enters the circulation and passes through the liver. This first-pass effect may explain the periportal iron accumulation in the hepatic lobule observed in iron overload disorders associated with inappropriately increased intestinal iron absorption^9^,^10^. Conversely, TF saturation is likely lower than average in plasma leaving the erythroid bone marrow, where most of the iron present is extracted for use by erythroid precursor cells. The erythroid bone marrow is the largest consumer of iron. Normally, two-thirds of the body iron endowment is found in developing erythroid precursors and mature red blood cells. Erythroid precursors express cell surface transferrin receptors (TfR1) that take up iron from holo-TF by receptor-mediated endocytosis^11^. Targeted disruption of the murine *TfR1* gene causes embryonic lethality, attributable to severe anemia^12^. Spontaneous mutations disrupting the *TF* gene demonstrate the importance of TF in animals 13 and humans 14. Lack of TF results in severe iron deficient mycrocytic anemia. However, iron deficiency occurs only in hematopoietic cells whereas other tissues develop massive iron overload. This underscores the importance of the TF-TfR1 endocytic cycle in erythropoiesis^11^, but further demonstrates that non-hematopoietic cells have alternative mechanisms to assimilate iron. It also highlights changes in overall iron homeostasis that result from iron deficient erythropoiesis. Hepcidin synthesis is markedly depressed and intestinal iron absorption is increased in congenital hypo-transferrinemia, apparently to try to compensate for the lack of iron available to erythroid precursors^13^,^15^,^16^. This is compelling evidence for a signaling mechanism that allows the erythroid bone marrow to communicate its iron needs to the intestine’s absorptive epithelium (see below).

### Iron absorption:

Iron in food is present as ferric iron or as heme. Heme is a biologically important iron containing compound and a key source of dietary iron but the mechanism by which the enterocyte takes up heme and catabolises it to utilise the iron is still poorly understood. Currently, there are two prevailing hypotheses explaining the mechanisms of this process; first, heme is taken up by receptor mediated endocytosis; secondly, the recent discovery of a heme transporter (PCFT/HCP1) that may have the capability of transferring heme from the small intestinal lumen directly into the cytoplasm^17^. One important criticism of the receptor mediated endocytosis hypothesis is that it assumes iron released from heme is transported out of the internalised vesicles in order to join the labile iron pool. Currently, no such transport process has been identified. However, it is possible that divalent metal transporter 1 (DMT1) may fulfil this role in a manner analogous to its role in the transferrin receptor cycle. In recent years, two mammalian heme transporters have been discovered, namely PCFT/HCP1^18^,^19^ and FLVCR^20^ possibly acting at the apical and basolateral site of the small intestinal enterocyte, respectively. At this early stage, however, the physiological relevance of these transporters to intestinal heme iron absorption is unclear. PCFT/HCP1 has been independently characterised as a folate/proton symporter and appears to play a key role in intestinal folate absorption. Interestingly, the folate transport capabilities of PCFT/HCP1 are at least an order of magnitude higher than that observed for heme, suggesting that folate may be the more physiologically relevant target of this transport protein. FLVCR is a heme exporter relevant for erythropoietic activity, that acts as an overflow valve for excess manufactured heme that would otherwise result in cellular toxicity. Intestinal cell line CaCo-2 does express the protein and it might be hypothesised it can exert similar function in intestinal cells too^21^.

Much more knowledge has been developed on non-haem iron absorption^4^,^17^. Non-haem iron requires to be converted in ferrous iron by the apical ferric reductase duodenal cytochrome B although the physiological significance of this pathway is the subject of continued debate. Following the reduction, iron crosses into the cytoplasm via an apical iron transporter, DMT1^22^. The physiological relevance of DMT1 in iron absorption, is confirmed in the Belgrade (*b*) rat and *mk* mouse which both exhibit a microcytic, hypochromic iron deficiency anaemia due to a G185R mutation to DMT1, resulting in a dramatic decrease in DMT1 function^23^.

### Cells regulating body iron homeostasis:

**Figure 2.**
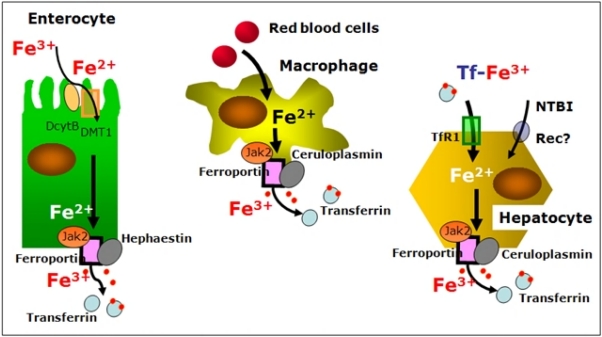
Cells regulating body-iron homeostasis. Enterocytes, macrophages and hepatocytes acquire iron from different sources and deliver it to the rest of the body through the iron exporter ferroportin, which needs copper-ferroxidases to release iron to plasma transferrin. Ferroportin acts under the control of hepcidin and this interaction can explain the systemic regulation of iron metabolism.

Besides enterocytes other cell lines, namely macrophages, hepatocytes in adults and placental cells during fetal life, have core functions within iron metabolism, that is to acquire iron from different sources (senescent erythrocytes and holotransferrin) and to deliver it to the rest of the body according to its needs (Figure 2). To do this important function these cells have a specialised mechanism for exporting iron to plasma, that is the iron exporter ferroportin^24^. Ferroportin needs copper-ferroxidases to release iron to plasma transferrin, namely hephaestin in duodenal cells^25^ and ceruloplasmin in hepatocytes, and macrophages^4^. When defective, these proteins induce cellular iron retention in specific cell types as shown in hephaestin deficient mice^26^ and in humans with aceruloplasminemia^27^. Ferroportin acts under the control of hepcidin and this interaction can explain the systemic regulation of iron metabolism^28^. Interestingly, it has been shown that hepcidin-induced internalization of ferroportin requires binding and cooperative interaction with Janus kinase (Jak2)^29^. Hepcidin binding to ferroportin results in the phosphorylation of ferroportin, a step necessary for its internalization by clathrin-coated pits and the kinase responsible for the phosphorylation is Jak2. This finding provides a mechanistic explanation for the dominant inheritance of hepcidin resistant ferroportin disease^30^ and suggests that Jak2 might represent an important link at the interface between erythropoiesis and iron metabolism.

### Iron storage:

Following phagocytosis of old and damaged erythrocytes, tissue macrophages, particularly in the spleen, lyse cells and catabolyse hemoglobin presumably by heme oxygenase, to liberate iron. Some iron remains stored in macrophages, although some is exported to plasma TF. Ferroportin is critical for macrophage iron export and can be regulated to change the ratio between stored and released iron^24^. Hepatocytes represent the main depot for iron storage in normal conditions and in non-transfusional iron overload. Although the TF cycle may be involved in hepatocyte iron acquisition to some extent, non–transferrin-bound iron (NTBI) uptake pathways become particularly important when serum iron levels exceed TF binding capacity^31^,^32^. The identity of the hepatocyte NTBI uptake system is not known, but DMT1 is unlikely to be involved, because hepatocytes can accumulate iron in the absence of DMT1^33^. Candidates for the NTBI transporter include Zip14, a member of the SLC39A zinc transporter family, which can mediate the uptake of zinc and NTBI in hepatocytes^34^. Hepatocytes have a large capacity to store excess iron. Most storage iron is probably in the form of ferritin, which can be mobilized when needed elsewhere in the body. Eventually, however, massive iron overload results in hepatotoxicity; hepatitis leads to fibrosis and cirrhosis. The liver is probably exposed to more NTBI than are other tissues because of the first-pass effect of the portal circulation. However, other tissues have NTBI uptake activities and load iron when NTBI is present in the plasma. The heart and endocrine tissues are particularly susceptible; cardiomyopathy and endocrinopathies are the predominant nonhepatic complications of iron overload. L-type calcium channels^35^ can mediate the uptake of NTBI in the myocardium, whereas other candidates, such as transient receptor potential canonical protein TRPC6^36^, need further confirm.

## Regulation of iron homeostasis

### Hepcidin: the master regulator

The liver peptide hepcidin regulates intestinal iron absorption and iron release from storage cells by binding ferroportin causing its internalization and degradation, thus exerting a general inhibitory effect on iron release in the body^28^,^37^,^38^. Animal models clarified the role of hepcidin as hepcidin knock-out mice developed massive iron overload^39^ and hepcidin over-expression induced iron deficiency 40. In physiological conditions, hepcidin production is tightly regulated in response to signals released from other organs, prevalently from bone marrow (erythroid regulator) and the iron stores (store regulator). Hepcidin levels increase in iron overload in order to limit iron absorption and are reduced up to undetectable levels in iron deficient erythropoiesis, either dependent from decreased iron supply or increased erythroid iron requirement, to allow iron acquisition^16^,^37^.

**Figure 3.**
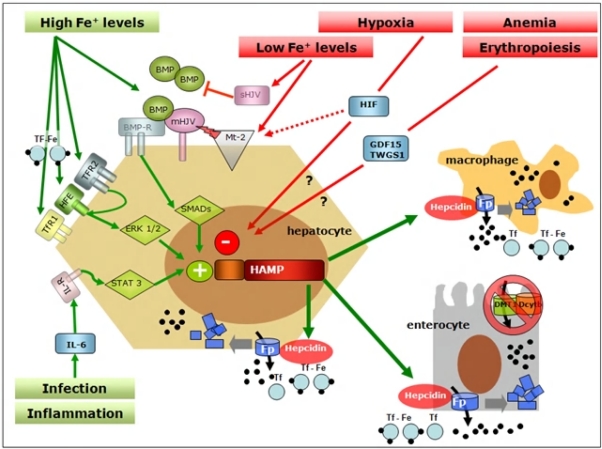
Signal pathways in systemic regulation of hepcidin. Many stimuli regulate expression of Hepcidin gene (HAMP) in the liver. One of the best known positive modulator is represented by Bone Morphogenetic Proteins (BMPs) that bind BMP-Receptor (BMP-R) on the surface of the hepatocyte resulting in SMAD-mediated induction of HAMP transcription. Hemojuvelin (mHJV) increases this signal acting as BMP co-receptor on the cell surface. In contrast, the soluble forms of HJV (sHJV), produced by HJV cleavage by furin at position 335, act as “decoy-receptor” competing with mHJV for the BMP ligand. Matriptase-2 (Mt2), which is activated by iron deficiency and by hypoxia, is the most potent inhibitor of hepcidin production by cleaving mHJV on hepatocyte surface and so preventing BMP-mediated hepcidin production. HAMP expression is also stimulated by inflammation, via the soluble mediator Interleukin-6 (IL6) and its specific membrane receptor (IL6-R) activating a STAT3-dependent signal pathway promoting HAMP transcription. HFE, TfR2 and TfR1 positively influence HAMP transcription in a ERK1/2 mediated way acting as a functional molecular complex on the cell surface playing a primary role in the hepatocyte sensing of circulating iron levels. Erythropoiesis, via the soluble mediator Growth Differentiation Factor 15 (GDF15) and Twisted Gastrulation (TWSG) 1, and hypoxia, via Hypoxia Inducible Factor (HIF), decrease HAMP expression (see text for further explanation).

Studies on genetic disorders of iron metabolism and of corresponding animal models have identified the hemochromatosis proteins (HFE, TFR2 and HJV) as the iron-dependent regulators of hepcidin expression. Patients affected by hemochromatosis have a defective synthesis of hepcidin that is absent in JH, reduced in type 3 HH due to TFR2 mutation or inadequate to the amount of iron overload in classical HH^38^. Such difference is related to the role of each protein in hepcidin regulation. HFE, TFR2 and HJV are all positive regulators and represent only a part of the complex regulation of hepcidin transcription, as summarised in Figure 3, where mediators and signalling pathways of iron-inflammatory- and erythroid- dependent regulation of hepcidin are reported. Recent studies suggest a role for HFE as a component of an iron-sensing complex that involves interactions with diferric transferrin, TFR1 and TfR2 at the plasma membrane of hepatocytes 41,42. Defective HFE or TFR2 prevents formation of a functional iron sensor and signal transduction effector complex leading to dysregulated hepcidin expression as observed in hereditary hemochromatosis. Indeed, double heterozygotes for HFE and TFR2 mutations develop hemochromatosis 43, whereas this does not occur in carriers of single allelic mutations in both HFE and HJV^44^,^45^, suggesting they behave to different pathways of hepcidin regulation. The main activator of hepcidin in iron overload is HJV, the protein mutated in type 2a juvenile hemochromatosis: HJV-deficient patients and mice have undetectable levels of hepcidin^46^,^47^. HJV is a GPI linked protein that activate hepcidin as a co-receptor for Bone morphogenetic proteins (BMPs), a family of cytokines that signal through SMAD proteins as a second messenger^48^. Several BMPs may activate hepcidin in vitro but the relevant BMP in vivo is BMP6, since knock out mice for this BMP have no developmental defect but develop severe iron overload^49^. That the BMPs pathway is involved in iron homeostasis through hepcidin regulation is further demonstrated by the severe iron overload and very low hepcidin expression reported in Smad4 liver conditional knock out^50^. Several other signals regulate hepcidin expression. Infection and inflammation markedly increase hepcidin synthesis through the IL-6/IL-6 receptor and STAT3 pathway, a mechanism largely implicated in the pathogenesis of anemia of chronic diseases^51^,^52^. Hepcidin inhibition occurs in iron deficiency, hypoxia and erythropoiesis expansion in order to increase iron export to plasma. Several inhibitors of hepcidin have been proposed: HIF-1a that is stabilised in hypoxia/iron deficiency, reduces hepcidin transcription by binding a HIF responsive element of hepcidin promoter^53^, the soluble variant of HJV downregulates hepcidin in vitro by competing with mHJV for the BMP ligand^54^, and matriptase 2 is a serin protease recently identified as a strong hepcidin inhibitor by cleaving membrane-HJV^55^. Accordingly, mutations in the gene coding matriptase 2 cause the rare form of iron refractory iron deficiency anemia in mice and humans^56^.

### Erythroid-dependent regulation of hepcidin:

**Figure 4.**
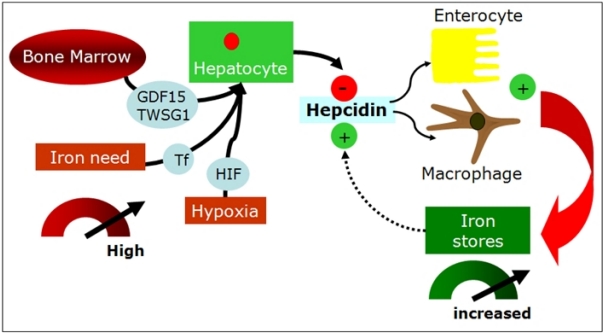
Hepcidin regulation by erythroid- iron- and hypoxia-related signals in iron-loading anemias. (GDF= growth differentiation factor; TWSG= twisted gastrulation; TF= holo-transferrin; HIF= hypoxia inducible factor)

In vivo studies demonstrated that the effect is predominantly due to the activation of erythropoiesis and to secondary changes in plasma and tissue iron^16^. This was demonstrated by Pak et al^57^ who showed that erythropoietin suppresses hepcidin synthesis, but that this effect was lost by pharmacologic inhibition of erythropoiesis. Similarly, Vokurka et al.^58^ showed a dramatic increase in hepcidin expression in mice when erythropoiesis was inhibited by irradiation or posttransfusion polycythemia. The nature of the erythropoietic regulator of hepcidin is now debated, but may include one or more proteins secreted by developing erythrocytes. When HepG2 cells were treated with sera from β-thalassemia patients, in which erythropoietic drive is greatly elevated, or with sera from HFE-hemochromatosis or control subjects, only β-thalassemia sera decreased hepcidin mRNA^59^. Another study treating HepG2 cells with sera from patients with iron-deficiency anemia or β-thalassemia showed similar results^60^. Regulation of hepcidin by erythropoietic activity is of particular importance in iron-loading anemias such as β-thalassemias and diserytropoietic anemias. Studies in animal models of thalassemia indicate that in conditions of extreme ineffective erythropoiesis, there is relatively little peripheral destruction of red cells and iron accumulates more rapidly in the liver than in the spleen, consistent with the interpretation that iron loading results primarily from increased intestinal absorption^59^,^61^. Thus, very high erythropoietic activity and increased iron requirement from the bone marrow generates a signal that can override hepcidin regulation by iron, leading to increased iron absorption, but the marrow signal that modulates hepcidin expression is debated (Figure 4). One proposed hepcidin inhibitor in these conditions is GDF15, a cytokine member of the TGF-β superfamily which is strongly expressed in hemoglobinized erythroblasts at the final stages of human erythropoiesis. GDF15 is hyperexpressed in thalassemia^62^, CDA type 1^63^ and, although at a lower level, in PKD and some myelodisplastic syndromes and it might mediate the bone marrow signal to the liver by partially suppressing hepcidin synthesis, as shown in hepatocyte cell colture^16^,^62^. Very recently, the same Authors, identified twisted gastrulation 1 (TWSG1) as a novel erythroid regulator of hepcidin expression in murine and human cells^64^. TWSG1 suppressed hepcidin in human hepatocytes through a BMP-dependent mechanism. It is proposed that TWSG1 and GDF15 act together to inappropriately inhibit hepcidin expression in thalassemia (Figure 4). Due to the expansion of erythropoiesis, TWSG1 expressed during the early stages of erythropoiesis acts indirectly by inhibiting BMP-mediated expression of hepcidin^64^. In thalassemia, over-expression of TWSG1 would inhibit the host’s ability to sense and respond to iron loading. It is also hypothesised that the increased expression of TWSG1 may also impact erythropoiesis in thalassemia by inhibiting the BMP4 dependent expansion of stress erythroblasts, which in turn would exacerbate the anemia.

### The role of hypoxi:

Several lines of evidence indicate that iron and oxygen homeostasis are tightly connected. Anemia generates tissue hypoxia and activates the cellular mediator of biological response to hypoxia: Hypoxia inducible transcription factors: HIFs. This induce a signalling cascade involving hundreds of genes. Seminal studies established an association between oxygen and iron regulation by showing that hypoxia results in higher iron absorption in mice and rats. Accordingly, both hypoxia and anemia induce the synthesis of erythropoietin (EPO) and are the two main signals that increase iron absorption independently of iron stores^1^,^65^. In addition, hypoxia was found to increase the expression of transferrin, transferrin receptor, ceruloplasmin and hemoxygenase-1 which are hypoxia-inducible HIF-1 target genes, thus enabling iron transport to erythroid tissue and cellular uptake, and iron recycling and release from stores.

Hepcidin is suppressed in human cultured hepatoma cells exposed to hypoxia^66^ and HIF-1 has been implicated in hypoxia-mediated hepcidin regulation^53^,^66^,^67^. HIF-1 binds to the hepcidin promoter in vitro and decreases is transactivation and stabilization of HIF in hepatocytes down-regulates hepcidin and increases ferroportin expression in mice. Thus, HIF-1 acts as a regulator of iron homeostasis, but recent studies also suggest a potential role of HIF-1α in iron sensing in the liver. In fact, iron deficiency increases HIF-1α levels in mice liver. In addition, one recent study demonstrates a role for HIF as a transcription factor that regulates the expression of divalent metal transporter (DMT)-1 and apical ferric reductase duodenal cytochrome b (Dcyt-b) genes, which are regulators of intestinal iron absorption^68^. Thus, HIF may act both as an iron sensor and iron regulator, and be an essential link between iron and oxygen homeostasis in vivo that through coordinate gene regulation mobilizes iron to support erythrocyte production. Other experimental studies further support the iron-oxygen connection, that involves iron deficient and hypoxia-dependent production of soluble-HJV^69^, GDF15 and Matriptase-2 in hepatocytes.

## Iron metabolism in thalassemia and sickle cell disease:

Patients affected by the most severe forms of thalassemia require chronic blood transfusions to sustain life and chelation therapy to prevent iron overload. Those affected by β-thalassemia intermedia do not require chronic blood transfusions but eventually develop elevated body iron loads due to ineffective erythropoiesis and hypoxia dependent hepcidin downregulation that, in turn, induces increased gastrointestinal iron absorption^16^,^61^,^70^. Iron absorption studies in patients affected by beta thalassemia intermedia show that the rate of iron loading from the gastrointestinal tract is approximately three to four times greater than normal^71^. In nontransfused patients with severe thalassemia, abnormal dietary iron absorption results in an increased body iron burden between 2 and 5 g per year depending on the severity of erythroid expansion^72^. If regular transfusions are required, as in β-thalassemia major patients, this doubles the rate of iron accumulation. In addition to the transfusion-related iron overload, increased iron absorption also plays a role in β-thalassemia major, in which its importance is inversely related to Hb levels^61^,^73^. As loading continues, the capacity of transferrin, the main transport protein of iron, to bind and detoxify this essential metal may be exceeded. The resulting nontransferrin-bound iron (NTBI) fraction within plasma may promote the generation of reactive oxygen species (ROS), propagators of oxygen-related damage^74^. Iron overload is responsible for the most damaging effects of the thalassemias, making iron chelation a focal point of the management of these diseases. In addition, as iron accumulates in the organs, dysfunction of the liver, endocrine glands, and heart become the main factors in limiting the survival of patients with β-thalassemia^2^.

Iron metabolism in SCD largely differs as compared to thalassemia. SCD is an inherited disorder of hemoglobin synthesis characterized by life-long hemolytic anemia, increased erythropoiesis and a chronic inflammatory state with endothelial activation and enhanced red cell and leukocyte adhesion^75^,^76^. There is no evidence of iron overload in non-transfused SCD patients, and iron deficiency may be even develop, possibly related to intravascular hemolysis (which constitutes about a third of the hemolysis in SCD), and the resulting excessive urinary losses of iron^77^. The variable clinical spectrum of SCD is the consequence of multiple events and genetic susceptibility that goes beyond the occurrence of a single amino acid substitution in the beta globin chain of hemoglobin. SCD is as much a disease of endothelial dysfunction as it is a hemoglobinopathy that triggers erythrocyte polymerization. Hemolytic rate is associated with a growing list of clinical complications of SCD that fall into two partially overlapping subphenotypes: a viscosity–vaso-occlusion phenotype versus one of hemolysis-endothelial dysfunction. The viscosity-vaso-occlusion subphenotype is associated with a lower hemolytic rate, marked by a higher hemoglobin level, and low plasma hemoglobin, lactate dehydrogenase, bilirubin and arginase levels. Patients with these features have a higher incidence of vaso-occlusive pain crises, acute chest syndrome, and osteonecrosis. In contrast, patients with the hemolysis-endothelial dysfunction subphenotype exhibit markers of high hemolytic rate, including low hemoglobin level, high plasma hemoglobin, LDH, bilirubin, and arginase, culminating in low nitric oxide bioavailability and high prevalence of pulmonary hypertension, leg ulceration, priapism, and stroke. Erythropoiesis is variably increased, but is not defective and nontransfused SCD patients do not spontaneously develop systemic iron overload, however, microvascular occlusion, erythrocyte sequestration and splenic infarction cause splenic iron overload^78^. In contrast, in nontransfused thalassemic patients the spleen is enlarged but not iron-loaded because of the lack of transfusions and because low hepcidin levels favor iron mobilization from reticuloendothelial stores^78^,^79^. Chronic transfusion therapy partially normalize many of the disparities between the diseases. In SCD patients, chronic transfusion therapy lowers the percent sickle hemoglobin to below 30%, dramatically decreasing intravascular hemolysis, splenic infarction, and the classic SCD vascular phenotypes associated with hemolysis nitric oxide dysregulation^75^,^80^. Patients with β-thalassemia and chronically transfused patients with SCD develop severe iron overload with iron deposition in liver, heart, spleen, and endocrine organs^2^,^81^. Although having similar transfusional burdens and somatic iron stores, patients with chronically transfused SCD appear to be at lower risk for endocrinopathy, cardiac dysfunction, iron-mediated oxidative stress, and extrahepatic iron deposition^2^. Risk for hepatic fibrosis during chronic transfusion therapy for SCD occurs at a HIC level 2.7-fold higher than historically seen in thalassemia 81. Transferrin saturation and circulating nontransferrin bound iron levels are lower in transfused patients with SCD relative to patients with TM having comparable somatic iron stores, suggesting disease-specific iron handling^2^,^74^. Some early reports link NTBI to iron-related cardiac disease and may explain the prevalence of severe heart disease in thalassaemia with their higher levels of NTBI compared to almost no cases in transfused SCD^2^. It has been postulated that the greater systemic inflammation observed in transfused patients with SCD may limit reticuloendothelial iron export and iron absorption through hepcidin or other iron mediators, but this hypothesis has never been proven^2^,^74^. Recent data indicate that, despite transfused SCD patients exhibiting heavy total body iron burden, they are monitored on a less frequent basis for iron-related organ damage compared to thalassemia patients. The reasons for these discrepancies are multifactorial and also likely include the perceived importance of the procedure by either the practitioner or the patient. Patients with SCD have far more acute and unpredictable clinical crises compared to patients with thalassemia; therefore, iron risk assessment is not perceived as having high priority in clinical care management. What is clear from these preliminary baseline data is that the disparity in the care of SCD patients who receive transfusions requires further study and that guidelines for the assessment of this select group of patients should be supported by existing evidence and expert opinion, until the full effect of iron overload in this population is better understood.

### Hepcidin levels in thalassemia and SCD patients:

Thalassemia intermedia and major are the most studied human models of hepcidin modulation by ineffective erythropoiesis alone and the combined and opposite effect of both ineffective erythropoiesis and transfusion dependent iron overload, respectively. Regular transfusions, in fact, induce massive iron loading but also inhibit erythropoietic drive. Accordingly, hepcidin production is higher in thalassemia major than in thalassemia intermedia although still inappropriate to the massive transfusional iron loading that partially counteracts the erythropoietic-dependent hepcidin downregulation^70^,^82^,^83^. Transfusions strongly influence hepcidin production as shown by the significant post-transfusion increase of urinary hepcidin levels in thalassemia patients, probably related to transfusion dependent suppression of erythropoiesis^84^. This result is also supported by Jenkins et al^85^ who found 5- to 8-fold increase in hepcidin mRNA in liver specimens obtained 1–3 days after red cell transfusion in patients with thalassemia major and SCD. This indicates that hemoglobin levels at the time of biopsy are a significant variable in determining liver hepcidin mRNA values.

Data on hepcidin levels in SCD are very limited and no data are presently available on GDF15 and TWSG1 expression. Kearney et al found significantly lower levels of urinary hepcidin in nine SCD patients when compared with normal controls^84^. In their analysis, hepcidin levels were negatively correlated with markers of erythroid proliferation, thus supporting the theory that hepcidin production is suppressed in scenarios of chronic haemolytic anemia and increased erythropoiesis. This finding, however, contrasts with the common knowledge that non-transfused patients with SCD do not develop iron overload. Although chronic hemolysis is one of the main features of SCD, the pathophysiology of SCD is not entirely red-cell related however, as vaso-occlusive crises and resultant tissue damage have been shown to result in chronic inflammation^74^,^76^, a condition that might influence hepcidin production^51^,^86^. In addition, transfusional iron overload might further increase hepcidin production. Thus, hepcidin levels in patients with SCD might be under the influence of different and contrasting stimuli, possibly leading to marked variability. Very recently Kroot et al 87 studied hepcidin and several serum parameters of erythropoietic, inflammatory and iron status in 16 patients with SCD. They found various serum parameters to vary widely within this population. In particular, they observed very high serum ferritin levels not simply related to the transfusion history and in the presence of normal transferrin saturation. This finding confirms that serum ferritin in SCD might not be suitable in the evaluation of hepatocyte iron loading and suggest an iron distribution pattern of the anemia of chronic disease, with relatively more iron in the RE system. The median serum and urine hepcidin-25 levels were similar for patients and controls. More precisely, serum hepcidin-25 levels were below the lower limit of detection in 5 SCD patients while in the rest they were within the normal range. Interestingly and not so easy to explain, the five patients with the lowest hepcidin levels did not show marked alterations of serum iron indices (transferrin saturation and serum ferritin ranged between 23–72%, and 40–213 μg/L, respectively). Results confirmed that also in SCD patients, erythropoiesis down-regulates hepcidin-25. In fact, when only sTfR was increased, serum hepcidin-25 levels were in the lower normal range or even not detectable. In cases where, together with a substantially increased sTfR, inflammation and/or high iron stores were also present, serum hepcidin-25 levels were in the normal range confirming the induction of hepcidin by inflammation and elevated iron stores in SCD patients.

## Conclusion:

Erythropoiesis and iron metabolism are inextricably linked. Hepcidin has a key role in coordinating iron procurement according to erythropoietic requirement. The recent tremendous advancement in the iron field have shed light on the various signals regulating hepcidin production and showed that erythroid-dependent regulation is absolutely prevailing. There are however, some issues to be clarified: a. if mediators other than GDF15 and TWSG1 exist and which are their interactions and mechanisms of hepcidin regulation; b. why, despite anemia and increased iron demand, and the evidence of reduced hepcidin synthesis, patients with haemolytic anemias do not develop the same amount of iron overload observed in the iron-loading anemias where ineffective erythropoiesis prevails on hemolysis. New links between erythropoiesis and iron metabolism have recently suggested: the link between Epo/EpoR/Jak2/Stat5 signalling and TfR1 mRNA transcription 88 and the role of Jak2 in the phosphorylation and recycling of ferroportin 29. In addition, based on preliminary studies in animal models of thalassemia, it has been suggested that use of Jak2 and analogous of hepcidin peptide might have a role in limiting ineffective erythropoiesis and increased iron absorption 73. Greater understanding on hepcidin regulation and function, and on the other molecules involved in the intricate association between iron and erythropoiesis, will shed light on new scenarios and will help develop more effective therapeutic options for disorders of iron homeostasis and erythropoiesis.
